# The effectiveness of an integrated care pathway in geriatric rehabilitation among older patients with complex health problems and their informal caregivers: a prospective cohort study

**DOI:** 10.1186/s12877-018-0971-4

**Published:** 2018-11-16

**Authors:** Irma H. J. Everink, Jolanda C. M. van Haastregt, Frans E. S. Tan, Jos M. G. A. Schols, Gertrudis I. J. M. Kempen

**Affiliations:** 1 Department of Health Services Research and Care and Public Health Research Institute (CAPHRI), Maastricht University, P.O. Box 616, 6200 MD Maastricht, The Netherlands; 2 Department of Methodology and Statistics and Care and Public Health Research Institute (CAPHRI), Maastricht University, P.O. Box 616, 6200 MD Maastricht, The Netherlands; 3 Department of Family Medicine and Care and Public Health Research Institute (CAPHRI), Maastricht University, P.O. Box 616, 6200 MD Maastricht, The Netherlands

**Keywords:** Aged, Geriatric rehabilitation, Subacute care, Integrated care, Pathway, Dependence in activities of daily living, Informal care

## Abstract

**Background:**

To improve continuity and coordination of care in geriatric rehabilitation, an integrated care pathway was developed and implemented in The Netherlands. The purpose of this study was to assess the effects of this pathway on patients and informal caregivers.

**Methods:**

Two cohorts of patients and their informal caregivers were prospectively recruited before implementation of the pathway (2011–2012) and after implementation of the pathway (2013–2014). Primary outcome measures were dependence in activities of daily living in patients (KATZ-15) and self-rated burden among informal caregivers (SRB-VAS). Secondary outcome measures were the frequency of performing extended daily activities, social participation, psychological well-being, quality of life and discharge location (patients) and quality of life and objective care burden (informal caregivers). Outcomes were measured at baseline, after three and after nine months.

**Results:**

No effect was shown on the KATZ-15 after three and nine months. However, a larger percentage of patients were discharged home in the care pathway cohort (83% vs 58.1% after three months and 88.6% vs 67.4% after nine months; *p* = 0.004). Furthermore, after three months, patients from the care pathway cohort performed more extended daily activities (*p* = 0.014) and informal caregivers experienced a lower self-rated burden (*p* = 0.05). After nine months, these effects disappeared. No differences were found for the other outcome measures.

**Conclusions:**

Due to the positive effects of the integrated care pathway, we are inclined to recommend implementing the care pathway in regular care. To have longer lasting effects among patients and informal caregivers, we suggest actively disseminating information about the pathway to primary care providers who are currently still unaware of its content.

**Trial registration:**

ISRCTN90000867 (date of registration: 07-04-2016).

## Background

Functional decline and deterioration in self-care abilities are common consequences of hospitalization among older adults, and can be exacerbated by inactivity and immobility during hospital stay [1, 2]. As a result, after hospital admission a considerable number of community-living older patients are discharged to an inpatient geriatric rehabilitation facility where they receive short-term multidisciplinary care to restore functional independence, such as activities of daily living (ADL), to improve quality of life and to prepare them to return to their former living situation [3].

A meta-analysis revealed that geriatric rehabilitation has beneficial effects on functional status and prevents permanent admission to nursing homes [4]. However, the fact that patients transfer between care settings (hospital, geriatric rehabilitation facility and primary care) and are confronted with multiple caregivers, forms a challenge for the coordination and continuity of care [5]. Frequently mentioned problems in these transitional phases are care plans not being communicated from one organization to the other, the transfer of medication lists which are not up-to-date or incomplete, and lack of communication between professionals from different organizations [6–10]. Furthermore, patients and their informal caregivers are often not sufficiently prepared for the transition to the home situation [5]. These problems in continuity of care could result in adverse events among patients, such as insufficient functional improvement, unnecessary hospital readmissions and permanent admission to a nursing home [6, 8, 10]. Moreover, care transitions affect the emotional, social, financial and physical functioning of informal caregivers. Therefore, inadequate care transitions are a substantial risk factor for high informal caregiver burden [11].

Various transitional care interventions have been developed to deal with these problems; these interventions focus mainly on discharge planning and discharge support for older adults. A systematic literature review by Laugaland and colleagues showed that the majority of these programs have beneficial effects, but that most interventions focus on single groups of caregivers, such as nurses or occupational therapists. Furthermore, all studies in this review focused on discharge interventions from hospital to home and did not include transfer to post-acute care settings, such as geriatric rehabilitation facilities [12].

To deal with challenges in continuity and coordination of care for patients who go through the trajectory of hospitalization, admission to a geriatric rehabilitation facility and discharge back to the home situation, an integrated care pathway in geriatric rehabilitation was developed and implemented in an urban region in the southern part of the Netherlands [13]. Integrated care pathways describe a sequence and timing of activities or interventions performed by care providers to obtain clinical goals. They comprise detailed information about which professional is responsible for these interventions and activities [14]. The integrated care pathway in geriatric rehabilitation focused on improving communication, triage and transfers of patients between hospital, geriatric rehabilitation facility and primary care organizations. To evaluate the effectiveness of this pathway, a prospective cohort study was conducted with a usual care cohort and a care pathway cohort of patients and informal caregivers. This study assessed the effectiveness of this pathway in comparison with usual care with respect to the level of dependence in activities of daily living among patients and the self-rated burden among informal caregivers as primary outcomes. Furthermore, recent performance of extended daily activities, social participation, psychological well-being, quality of life and discharge location were assessed as secondary outcomes among patients, and, among informal caregivers, quality of life and objective care burden.

## Methods

### Study design

A prospective cohort study was used to assess the effects of the integrated care pathway. Two cohorts of patients and informal caregivers were prospectively recruited in the geriatric rehabilitation facility where the pathway was implemented. This geriatric rehabilitation facility was situated in the Maastricht area (in the southern part of the Netherlands). The first cohort of patients and informal caregivers (the care as usual cohort) was included in the period April 2011 – March 2012, prior to implementation of the care pathway. The second cohort (the care pathway cohort) was included in the period April 2013–August 2014, after implementation of the pathway. This study design and methods were approved by the Medical Ethics Committee of University Hospital Maastricht (#11–4-020).

### Participants

The participants of this study were patients admitted to a geriatric rehabilitation facility in Maastricht, the Netherlands, and their informal caregivers. In the Netherlands, patients admitted to a geriatric rehabilitation facility are categorized into four groups: patients with stroke, trauma orthopedics, elective orthopedics and the residual, referred to as patients with complex health problems. The pathway described in the present study was developed for this heterogeneous group of patients with complex health problems. These patients often suffer from multi-morbidity, mostly involving cardiac problems, problems with the respiratory system, neurological problems, oncological problems and other internal medicine problems such as gastrointestinal problems. Disease exacerbations are common in this group, leading to hospital readmissions and the necessity for geriatric rehabilitation. All patients from this group were eligible for participation if they were admitted to the geriatric rehabilitation facility in the period April 2011–March 2012 or in the period April 2013–August 2014, aged ≥65 years, admitted to the hospital prior to admission to the geriatric rehabilitation facility and were community-dwelling prior to hospital admission. Patients in the two cohorts were not eligible to participate if the elderly care physician assessed their cognitive status as insufficient for participation, based on their expert opinion.

Informal caregivers were recruited by asking the included patients who their main informal caregiver was, and whether they permitted the researchers to invite them for participation in the study. Informal caregiving was defined as voluntary and unpaid care, delivered on a structural basis to people with physical, cognitive of mental deficiencies. This could be either a family member or not. If the patient approved contacting their informal caregiver, the latter was invited for participation by telephone. All patients and informal caregivers provided written informed consent.

### Intervention

The integrated care pathway was developed by reviewing relevant literature and consulting experts. Furthermore, iterative meetings with two multidisciplinary workgroups of professionals and one workgroup of patients and informal caregivers were organized. During these meetings, current practice, barriers to and incentives for change were analyzed and proposals for improving the care process were generated. These proposals for improvement were critically discussed in the multidisciplinary workgroups, finally resulting in the integrated care pathway. The development and implementation process of the integrated care pathway is described in more detail elsewhere [13]. Due to the heterogeneity of this group of patients with complex health problems, the pathway is focused on the process of care instead of the contents of the rehabilitation treatment and involves the full trajectory of hospital admission, discharge to the geriatric rehabilitation facility and discharge back to the community. The key components of the pathway are the following:

1. A care pathway coordinator is appointed. The role of the care pathway coordinator is to act as a contact person for professionals involved in the pathway, to further streamline the care processes in the pathway and improve continuity and coordination of care.
2. A triage instrument is used by discharge nurses in the hospital. The instrument instructs discharge nurses to gather information for potential patients for geriatric rehabilitation about functional prognosis, endurability of the patient, teachability/trainability and patients’ and informal caregiver’s needs and abilities. This information should enable the nurses to decide if geriatric rehabilitation is indeed appropriate for a patient or not. If the discharge nurse has doubts about the appropriateness of geriatric rehabilitation for a patient, the elderly care physician from the geriatric rehabilitation facility is consulted, and makes the final decision.
3. Patients and their informal caregivers are always actively involved in the triage decision in the hospital, and in the establishment of their care and treatment plan in the geriatric rehabilitation facility and in primary care;
4. All patient discharge summaries (medical, nursing and from allied care professionals) from the hospital to the geriatric rehabilitation facility and from the geriatric rehabilitation facility to primary care are sent on the day of discharge and are of high quality (clear andcomprehensive);
5. Meetings between care professionals from the hospital and the geriatric rehabilitation facility are organized at least twice per year, and between the geriatric rehabilitation facility and primary care organizations at least once per year. These meetings focus on improving the triage process, the timing and quality of discharge summaries and the transfer of patients between the hospital, geriatric rehabilitation facility and primary care organizations.

The agreements in the care pathway can be retrieved in the Appendix.

As the integrated care pathway was regular care from April 2013 onwards in the participating geriatric rehabilitation facility, all patients in the group of complex health problems admitted after April 2013 received care according to the pathway. In the care as usual cohort, there was no care pathway coordinator appointed, the decision to refer someone to the geriatric rehabilitation facility was made without the use of an official triage instrument and there were no structural meetings between professionals of the hospital, the geriatric rehabilitation facility and the primary care organizations. Furthermore, the active involvement of patients and informal caregivers in their rehabilitation trajectory and the timeliness and high quality of discharge summaries were not established in agreements or protocols in the care as usual cohort.

### Outcome measures

The primary outcome measure used to evaluate the effects of the pathway on patients was dependence in activities of daily living, measured with the Katz Index KATZ-15 [15]. This scale assesses one’s ability to perform activities of daily living by asking 15 questions related to the (instrumental) activities of daily living, self-care and mobility. Each question could be answered with “no help needed” (0) or “help needed” (1) and a total score of 15 could be achieved. A higher score represents more dependence in activities of daily living.

Five secondary outcome measures were used to assess the effects of the pathway on the patients. The first secondary outcome measure was recent performance of extended daily activities, measured with the Frenchay Activities Index (FAI) [16]. This index consists of 15 items assessing the frequency with which activities are performed that reflect the extended activities of daily life. These activities range from domestic chores, to leisure and outdoor activities. The frequency of performing these activities can be scored on a scale ranging from “never” (1) to “often” (4). The second secondary outcome measure was social participation, assessed using two subscales of the Impact on Participation and Autonomy (IPA) questionnaire [17]. These subscales are “autonomy outdoors” and “social life and relationships” and consist, respectively, of 5 and 7 items. The questions examine the extent to which people are able to perform activities such as visiting friends and going on a trip or holiday whenever they want to (autonomy outdoors) and the degree to which they are able to interact with people on an equivalent level (social life and relationships). Answer options range from “very good” (1) to “poor” (5). Whereas the KATZ-15 thus mainly focuses on self-care and mobility, the FAI adds somewhat more complex leisure and outdoor activities. Finally, the IPA also focuses on interaction with other people. The third secondary outcome measure was psychological well-being, measured using a subscale from the RAND-36 item Health Survey (RAND-36) [18]. This subscale consists of five items focusing on feelings (such as happiness, sadness and nervousness) people experienced in the last month. These items have six answer categories each, ranging from “always” (1) to “never” (6). The fourth secondary outcome measure was quality of life, measured with a modified version of Cantril’s Self Anchoring Ladder (CSAL) [15]. This measure asks patients to value their quality of life on a scale ranging from 0 to 100, with higher scores indicating a better quality of life. The fifth and last secondary patient outcome measure assessed was discharge location after inpatient geriatric rehabilitation. This was scored as back home (0) or not back home (1), the latter including institutionalization (admission to an elderly care home, a nursing home or palliative care in a hospice), hospital readmission or death.

The primary outcome measure used to assess the effects of the pathway on informal caregivers was self-rated burden of informal caregiving, measured with the Self-Rated Burden Visual Analogue Scale (SRB-VAS). The self-rated burden VAS assesses on a scale from 0 to 10 how burdensome informal caregiving is for the informal caregiver, with a higher score indicating a higher burden [19].

Secondary outcomes used to assess the effects of the pathway on informal caregivers were quality of life, assessed with a modified version of Cantril’s Self Anchoring Ladder [15], and objective burden of caregiving, measured using the Erasmus iBMG instrument [15]. This instrument asks informal caregivers how many hours per week they spend on various caregiving tasks.

### Data collection

Primary and secondary outcome measures for this effect evaluation were collected through structured face-to-face interviews by a trained research assistant with patients, and, for informal caregivers, through written questionnaires. The interviews with patients were conducted at admission in the geriatric rehabilitation facility (baseline), after 3 months and after 9 months. The written questionnaires were sent to the informal caregivers using the same timeframes. Discharge location was assessed by reviewing patient files in the geriatric rehabilitation facility.

### Statistical analysis

Statistical analyses were performed using the statistical software package SPSS for Windows, version 22. Descriptive statistics, independent t-tests and chi square tests were used to describe and compare the baseline characteristics of patients and informal caregivers in the two cohorts. Because data was collected longitudinally, a two-level mixed model was used to compare the two cohorts of patients and informal caregivers with respect to the continuous primary and secondary outcome measures. Repeated measurements were the first level observations and respondents were the second level observations. A longitudinal model was specified with the outcome variable as a function of all three time points treated as dependent. Adjusted mean differences were calculated to express the differences between groups and were fully corrected for baseline differences (by specifying group and time as main effects as well as the interaction between time and group). The group differences were also corrected for age, sex, living situation (not living alone vs. living alone), educational level (lower than vocational school vs. vocational school or higher), multi-morbidity (one condition vs. the presence of two or more conditions). As the secondary outcome measure “discharge location” is dichotomous (home vs. not home), this outcome measure was analyzed with a standard logistic regression model. The previously mentioned covariates for patients (i.e. age, sex, living situation, educational level, and multi-morbidity) were also included in this model.

For the informal caregivers, covariates included in each multilevel model were age, sex and living situation (not living with care receiver vs. living with care receiver). Adjusted mean differences were calculated to express the differences between groups, fully corrected for baseline differences. Missing data among patients and among informal caregivers were assumed to be missing at random.

## Results

### Patients

**Fig. 1:**
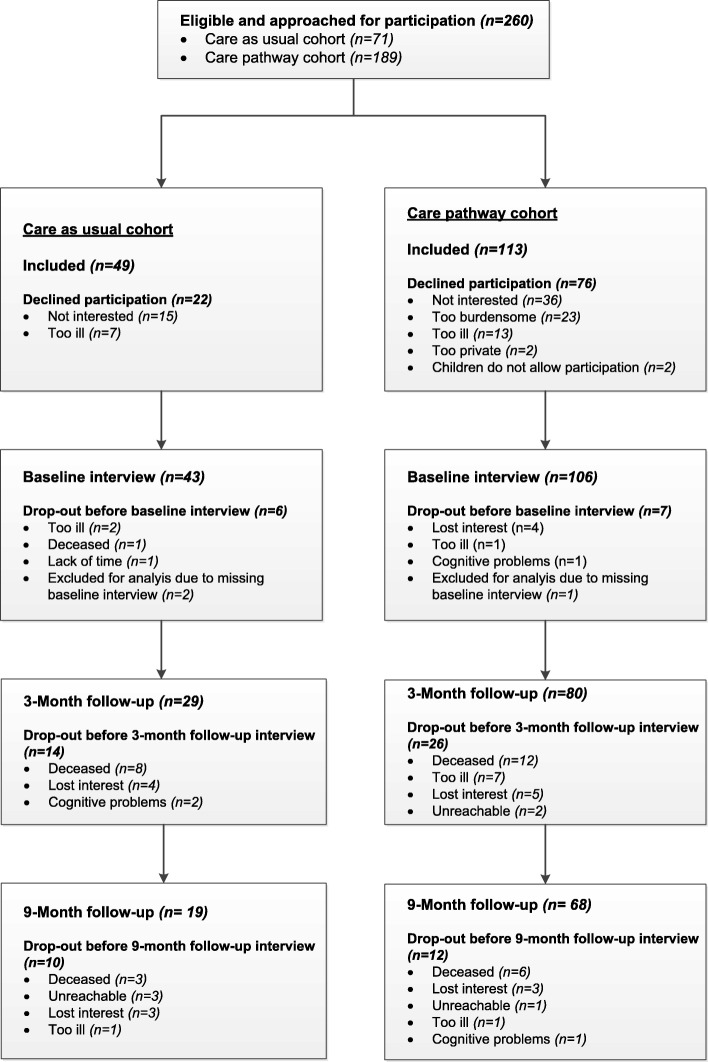
Flowchart of Patients through the Study

In total, 260 patients in the two cohorts were eligible for participation in the study: 71 in the care as usual cohort and 189 in the care pathway cohort. In the care as usual cohort, 49 patients agreed to participate (69%) and in the care pathway cohort this number was 113 (60%). The reasons for not participating were rather similar in both cohorts. Figure 1 shows the flowchart of the patient study population.

All interviews with patients were performed face-to-face. Because six patients in the care as usual cohort and seven patients in the care pathway cohort did not participate in the baseline measurement (because they dropped out of the study prior to their first interview or had only follow-up measurements), these patients were not included in the analyses. Thus the total number of patients in the analyses was 43 and 106, respectively. Total dropout during the course of the study in the care as usual cohort was 24 (56%) and 38 (36%) in the care pathway cohort. The reasons for dropout are provided in Fig. 1 as well.

Table 1 shows the baseline characteristics of the patients. More patients in the care pathway cohort suffered from diseases of the locomotor system in comparison with the care as usual cohort (18.1% versus 4.7%). Furthermore, the difference between the two groups in the number of patients suffering from cardiovascular diseases is borderline significant (37.2% in the care as usual cohort versus 22.9% in the care pathway cohort; *p* = 0.074).There are no other statistically significant differences between the two cohorts.

**Table 1:** Baseline Characteristics of Patients in Both Cohorts

Characteristics	Care as usual cohort *n* = 43	Care pathway cohort *n* = 106	*p*-value
Mean age (sd)	79.6 (7.1)	80.7 (6.9)	0.370
Sex (% female)	65.0%	67.9%	0.471
Living situation (% living alone)	67.4%	68.9%	0.865
Education (% ≥ vocational school)	60.5%	67.9%	0.385
Multi-morbidity (% having at least 2 conditions)	87.8%	88.7%	0.882
Medical diagnosis			
• Cardiovascular diseases (n, %)	16 (37.2%)	24 (22.9%)	0.074
• Internal medicine diseases (n, %)	15 (34.9%)	30 (28.6%)	0.449
• Oncological diseases (n, %)	5 (11.6%)	7 (6.7%)	0.331
• Respiratory diseases (n, %)	4 (9.3%)	11 (10.5%)	0.547
• Diseases of locomotor system (n, %)	2 (4.7%)	19 (18.1%)	0.033^†^
• Neurological diseases (n, %)	1 (2.3%)	8 (7.6%)	0.448
• Other (n, %)	0 (0%)	6 (5.7%)	0.181
Primary outcome measure			
Dependence in activities of daily living (mean score KATZ-15; range 0–15*) (sd)	6.6 (3.7)	5.7 (3.3)	0.179
Secondary outcome measures			
Extended daily activities (mean score FAI; range 15–60) (sd)	33.5 (9.6)	32.2 (8.7)	0.411
Social participation (mean score IPA; range 12–60) (sd)	30.5 (6.5)	29.2 (6.2)	0.310
Psychological well-being (mean score subscale RAND-36; range 5–30) (sd)	21.1 (6.2)	21.8 (5.6)	0.481
Quality of life (mean score CSAL; range 1–100) (sd)	66.4 (12.9)	65.6 (14.8)	0.768

Internal medicine diseases are diseases of the internal organs such as renal diseases, gastrointestinal diseases and infections

*KATZ-15* modified version of the Katz Index of Independence in Activities of Daily Living, *FAI* Frenchay Activities Index, *IPA* Impact on Participation and Autonomy, *CSAL* Cantril’s Self Anchoring Ladder

^a^The underlined score represents the most favorable score

^†^Statistically significant (*p*-value < 0.05)

### Informal caregivers

In total, 26 informal caregivers were included in the care as usual cohort and 28 informal caregivers in the care pathway cohort. In the care as usual cohort, 9 patients (20.9%) indicated not having an informal caregiver. Furthermore, 9 informal caregivers did not participate because the person they cared for died (*n* = 4) or they were not interested in participating (*n* = 4). In the care pathway cohort, the main reasons for not participating were that (a) the patient indicated not having a caregiver (*n* = 32), (b) the caregiver was not interested in participating (*n* = 24), or (c) the patient did not want to burden the informal caregiver (*n* = 10).

In Table 2, the background characteristics of the informal caregivers are displayed. In the care as usual cohort, two informal caregivers participated only in follow-up measurements, and in the care pathway cohort, six informal caregivers participated only in follow-up measurements. Therefore, these informal caregivers did not have scores on the primary and secondary outcome measures at baseline. As shown in Table 2, the differences in baseline characteristics between the two groups at baseline are not statistically different.

**Table 2:** Baseline Characteristics of Informal Caregivers in Both Cohorts

Characteristics	Care as usual cohort *n* = 26	Care pathway cohort *n* = 28	*p*-value
Mean age (sd)	58.9 (14.4)	61.3 (13.9)	0.537
Sex (% female)	20 (76.9%)	20 (71.4%)	0.645
Living together with patient (%)	8 (30.8%)	5 (17.9%)	0.267
Primary outcome measure	n = 24	*n* = 22	
Self-rated burden of informal caregiving (mean score SRB-VAS; 0-10^a^) (sd)	4.7 (2.8)	5.5 (2.5)	0.289
Secondary outcome measures			
Quality of life (Mean score CSAL; range 0-100^a^) (sd)	70.9 (13.8)	71.0 (12.1)	0.991
Mean (sd) objective burden of caregiving (Erasmus iBMG)			
• Domestic duties (hours/week)	6.1 (9.3)	7.7 (13.6)	0.654
• Personal care (hours/week)	1.7 (4.3)	0.17 (0.76)	0.121
• Moving outside the house (hours/week)	4.2 (3.8)	5.3 (4.0)	0.401
• Number of hours help of other informal caregivers / volunteers (hours/week)	1.6 (2.8)	1.1 (2.3)	0.610

*SRB* Self-Rated Burden visual analogue scale, *CSAL* Cantril’s Self Anchoring Ladder

^a^The underlined score represents the most favorable score

In the care as usual cohort, 10 informal caregivers dropped out during the study due to variable reasons: the patient died (*n* = 6), the informal caregiver did not return the questionnaire (*n* = 3) or lost interest (*n* = 1). In the care pathway cohort, 14 informal caregivers dropped out for the following reasons: the patient died (*n* = 7), the caregivers indicated they no longer had informal care tasks (*n* = 3), they simply did not return the questionnaire (*n* = 2), they lost interest (*n* = 1), or were too ill (*n* = 1).

### Effects of the integrated care pathway on patients

The mixed model analysis showed no difference after 3 and 9 months in the primary outcome measure, dependence in activities of daily living as measured with the KATZ-15. The adjusted mean difference was − 0.51 (*p* = 0.360) after 3 months and − 0.14 (*p* = 0.862) after 9 months (Table 3). Furthermore, a significant adjusted mean difference of 4.14 (*p* = 0.014) was found for the secondary outcome measure as measured with the FAI – the frequency of performing extended daily activities - after 3 months. This significant difference disappeared after 9 months (adjusted mean difference = 1.84, *p* = 0.288). No significant differences were found for social participation, psychological well-being and quality of life after 3 and 9 months.

**Table 3:** Multilevel Analysis for Differences between Patients in the Two Cohorts at 3 and 9-Month Follow-up (*n* = 149)

	3-month follow-up Mean (SD) ^a^		Adj. mean difference^b^ (95% CI)	*p*-value	9-month follow-up Mean (SD) ^a^		Adj. mean difference^b^ (95% CI)	*p*-value
Primary outcome measure	CUC; *n* = 26	CPC; *n* = 75	*n* = 147		CUC; *n* = 19	CPC; *n* = 68	*n* = 147	
Dependence in activities of daily living (KATZ-15; range 0-15^c^)	5.7 (2.8)	4.6 (2.4)	−0.51 (−1.60, 0.59)	0.360	5.0 (3.0)	4.4 (2.9)	−0.14 (− 1.41, 1.12)	0.862
Secondary outcome measures								
Extended daily activities (FAI; range 15–60)	27.4 (9.7)	31.1 (9.4)	4.14 (0.86, 7.42)	0.014†	29.4 (11.2)	31.0 (9.4)	1.84 (−1.58, 5.26)	0.288
Social participation (IPA; range 12–60)	31.0 (6.2)	28.9 (6.8)	−1.20 (−4.28, 1.88)	0.441	30.8 (8.0)	30.8 (8.3)	−0.27 (−4.70, 4.16)	0.903
Psychological well-being (Subscale RAND-36; range 5–30)	22.8 (5.0)	23.7 (4.7)	−0.53 (−2.61, 1.54)	0.610	22.8 (6.3)	22.9 (5.6)	−0.91 (−3.67, 1.94)	0.529
Quality of life (CSAL; range 0–100)	67.9 (14.1)	70.7 (9.4)	4.95 (−2.17, 12.08)	0.171	71.4 (9.2)	68.9(16.4)	1.54 (−7.29, 10.37)	0.730

*CUC* Care as Usual Cohort, *CPC* Care Pathway Cohort, *KATZ-15* modified version of the Katz Index of Independence in Activities of Daily Living, *FAI* Frenchay Activities Index, *IPA* Impact on Participation and Autonomy, *CSAL* Cantril’s Self Anchoring Ladder

†Statistically significant (*p*-value < 0.05)

^a^Unadjusted means

^b^Adjusted for age, sex, living situation, educational level, multi-morbidity and the interaction term “group*time”

^c^The underlined score represents the most favorable score

As shown in Table 4, a significantly higher proportion of patients in the care pathway cohort were discharged to their home situation compared to patients in the care as usual cohort. This difference between the two cohorts is visible after 3 months (83.0% vs 58.1%; *p* = 0.004) as well as after 9 months (88.6% vs 67.4%; *p* = 0.004).

**Table 4:** Discharge Location after Geriatric Rehabilitation at 3 and 9 Month Follow-up (*n*=149)

	3 months follow-up			9 months follow-up		
	CUC *n* = 43	CPC *n* = 106	*P*-value^*^	CUC *n* = 43	CPC *n* = 106	*P*-value^*^
Home	25 (58.1%)	88 (83.0%)	0.004†	29 (67.4%)	94 (88.6%)	0.004^†^
Not home	18 (41.9%)	18 (17.0%)		14 (32.6%)	12 (11.4%)	

*CUC* Care as Usual Cohort, *CPC* Care Pathway Cohort

^*^Adjusted for age, sex, living situation, educational level and multi-morbidity

^†^Statistically significant (p-value < 0.05)

### Effects of the integrated care pathway on informal caregivers

As shown in Table 5, after 3 months there was a significant adjusted mean difference for the primary outcome measure self-rated burden (SRB-VAS) among informal caregivers (− 1.54; *p* = 0.05). The significance of this difference disappeared after 9 months (*p* = 0.077). Table 5 shows that implementation of the integrated care pathway did not result in significant differences between the two cohorts of informal caregivers on the secondary outcome measures after 3 and 9 months (Table 5).

**Table 5:** Multilevel Analysis for Differences between Informal Caregivers in the Two Cohorts at 3 and 9-Month Follow-up (*n* = 54)

	3-month follow-up Mean (SD)^a^		Adj. mean difference^b^ (95% CI)	*p*-value	9-month follow-up Mean (SD) ^a^		Adj. mean difference^b^ (95% CI)	*p*-value
Primary outcome measure	CUC; *n* = 18	CPC; *n* = 19	*n* = 54		CUC; *n* = 16	CPC; *n* = 14	*n* = 54	
Self-rated burden of informal caregiving (SRB-VAS; 0-10^c^)	5.4 (2.2)	4.1 (2.4)	−1.54 (−3.08, − 0.00)	0.050^†^	4.4 (2.2)	3.5 (2.6)	−1.54 (−3.25, 0.17)	0.077
Secondary outcome measures								
Quality of life (CSAL; range 0–100)	68.2 (14.3)	73.2 (15.2)	3.11 (−3.86, 10.01)	0.371	68.7 (11.3)	73.2 (8.2)	5.26 (−2.24, 12.77)	0.158
Mean objective burden of caregiving (Erasmus iBMG)								
• Domestic duties (hours/week)	11.7 (20.9)	9.7 (14.0)	−3.15 (−13.14, 6.84)	0.525	10.4 (12.8)	9.1 (12.5)	−4.54 (−14.54, 5.46)	0.361
• Personal care (hours/week)	2.0 (3.9)	0.9 (2.4)	0.54 (−1.80, 2.87)	0.646	4.1 (10.5)	5.6 (12.4)	2.99 (−5.36, 11.33)	0.470
• Moving outside the house (hours/week)	3.2 (2.7)	3.8 (2.6)	−0.72 (−3.33, 1.90)	0.583	3.9 (4.2)	5.6 (8.1)	1.65 (−3.40, 6.71)	0.510
• Number of hours help from other informal caregivers / volunteers (hours/week)	1.9 (3.0)	1.0 (1.7)	−0.67 (−2.67, 1.32)	0.500	6.4 (20.8)	2.9 (4.8)	−1.92 (−11.73, 7.89)	0.684

*CUC* Care as Usual Cohort, *CPC* Care Pathway Cohort

^†^Statistically significant (*p*-value < 0.05)

^a^Unadjusted means

^b^Adjusted for age, sex, living situation and the interaction term “group*time”

^c^The underlined score represents the most favorable score

## Discussion

This study examined if implementation of an integrated care pathway in geriatric rehabilitation resulted in lower dependence in activities of daily living among patients and decreased self-rated burden among informal caregivers. The results of this study show that implementation of the pathway had no significant effect on level of dependence in activities of daily living among patients over a period of 3 and 9 months. A statistically significant effect was found for self-rated burden among informal caregivers after 3 months. However, this effect disappeared after 9 months. With respect to secondary outcome measures, our study showed that the pathway had a significant effect on the frequency of performing extended daily activities among patients after 3 months. This effect also disappeared after 9 months. No significant effect was found for the secondary outcome measures social participation, psychological well-being and quality of life among patients, or on quality of life and objective care burden among informal caregivers after 3 and 9 months. Therefore, it can be stated that overall, only small effects were found on the functional outcome measures. However, a significantly higher proportion of patients in the care pathway cohort were discharged back home in comparison with patients in the care as usual cohort. Overall, if patients are discharged home, their functional status is higher compared to patients who are still in the geriatric rehabilitation facility or patients who are admitted to nursing homes. This is also shown by the difference in score on the FAI after 3 months. Furthermore, in addition to the effects on these functional outcome measures, the economic evaluation executed alongside this study showed that implementation of the pathway resulted in a shorter length of stay in the hospital and the geriatric rehabilitation facility [20]. As a consequence, large cost savings were achieved.

It is noteworthy to mention that an effect was found on the secondary outcome measure performance of extended activities of daily living, while no effect was found on the primary outcome measure independence in activities of daily living. A reason could be that the integrated care pathway is focused on the active involvement of patients in the establishment of their rehabilitation goals. When rehabilitation goals are tailored and more personalized towards the patient’s wishes and preferences, patients will probably be better prepared to restart leisure and outdoor activities once discharged to the home situation. This might indicate that patients are taught how to resume these extended daily activities, irrespective of their limitations or level of dependence in activities of daily living. Another remarkable finding is that although more patients were discharged home in the care pathway cohort, the self-rated burden among informal caregivers after 3 months was significantly lower. This might indicate that patients are either less dependent on the informal caregivers, or the informal caregivers are better prepared at the moment the patients are discharged home.

The statistically significant favorable outcomes on ‘frequency of performing extended daily activities’ among patients and ‘self-rated burden’ among informal caregivers after 3 months, disappeared after 9 months. An explanation for the disappearance of these effects could be that the pathway is focused on patients who transfer between hospital, geriatric rehabilitation facility and home. After patients returned home, the integrated care pathway turns into regular primary care. This means that the reach of the pathway (i.e. the active involvement of the care pathway coordinator) extends to approximately 1 month after discharge of a patient from the geriatric rehabilitation facility. Thus, after 9 months, the pathway activities are no longer active. Still, it was expected that due to the improved transfer phases and improved coordination of follow-up care, the effects of the integrated care pathway would carry on for a longer period of time. Another explanation could be that the number and variety of professionals providing primary care in the Maastricht region is large (i.e. home care providers, general practitioners, physiotherapists, occupational therapists, etc.) and rather dispersed. This might have affected the extent to which multidisciplinary and coordinated care was provided in the home situation. Although we tried to reach all primary care providers via their professionals associations, it is possible that not all providers were aware of the content of the integrated care pathway. In addition it is possible that the care providers who were aware of the agreements of the pathway did not always act upon these agreements, due to lack of time, motivation or other hindering factors. Therefore, in the future it should be more closely monitored to which extent primary care professionals are actually aware of the pathway and have implemented its different components in daily practice. Targeted implementation strategies should then be deployed to improve the implementation of the care pathway in primary care.

Finally, a process evaluation executed alongside this study (described elsewhere) showed that this care pathway is a promising start, but there seems to be room for optimization as well [20].

Due to a lack of studies in the area of geriatric rehabilitation, it is not possible to compare our results to related studies within the domain. However, several studies concerning inter-organizational care pathways involving hospital and primary care showed positive results regarding care coordination, morbidity, drug-related adverse events, hospital readmission rates, emergency department visits and healthcare costs [21–25]. Patient-related outcomes such as dependence in activities of daily living and perceived burden of care for informal caregivers were not assessed in these studies.

Although this pathway was developed in the Netherlands, the majority of its content is relevant internationally as well. As an increasing number of older people suffer from multi-morbidity, they mostly receive care from a range of professionals in various organizations [22]. The principles of this pathway regarding inter-organizational collaboration and improved communication between providers can be used to facilitate continuity and coordination of care between these organizations.

Some limitations of our study should be mentioned. First, because the effects of the pathway were studied in a prospective cohort study where the care as usual cohort was included in 2011–2012 and the care pathway cohort was included in 2013–2014, the possible influence of external factors on the results has to be considered. Although the use of the triage instrument by discharge nurses in the hospital was a fundamental part of the integrated care pathway, the stricter admission criteria for geriatric rehabilitation enforced by this triage instrument were accompanied by the nationwide introduction of stricter admission criteria in 2013. These criteria were used to facilitate the development and implementation of the triage instrument. This has probably influenced the type of patients who were eligible for geriatric rehabilitation. Although the two cohorts were comparable on their baseline characteristics, there is a reasonable chance that selection bias occurred. This might explain (part of) the effect on discharge destination*.* Second, because our patient population was highly frail (as indicated by the fact that almost 20% of the patients died during the course of the study; Fig. 1), the number of patients included was relatively low and the number of dropouts was large. This is also true for the group of informal caregivers: many patients in the care pathway cohort stated that they were independent prior to hospital admission and therefore did not have an informal caregiver. This resulted in smaller numbers for inclusion which also could have resulted in the failure to detect an effect. Third, we do not have data of patients and informal caregivers who declined participation. Therefore, we cannot compare this group to our participants. Furthermore, selective dropout might have resulted in underestimation or overestimation of our results. However, because the reasons for non-participation and dropout are rather similar across cohorts we have no reason to assume that they are disproportionately related to the primary or secondary outcome measures. Furthermore, because multilevel analyses were performed, the risk of bias due to missing values decreased.

Our study has two important strengths as well. First, as an observational design was used to assess the effects of the pathway, there was room for optimization and adjustment of the pathway during the implementation phase based on the needs and circumstances of the organizations involved. Therefore, results of this evaluation can be interpreted as ‘real world’ results, which makes it more likely that results are generalizable towards other geriatric rehabilitation settings. Furthermore, because thorough research into the effects of integrated care pathways across organizational and disciplinary borders is scarce [26], this study forms a unique and valuable contribution to existing knowledge in the complex domain of integrated care pathways and geriatric rehabilitation care.

## Conclusions

We conclude that implementation of the integrated care pathway resulted in a significantly higher proportion of patients being discharged to the home situation after geriatric rehabilitation. Furthermore, the frequency of performing extended daily activities among patients in the care pathway cohort was significantly higher after 3 months compared to patients in the care as usual cohort, and the self-rated burden of informal caregivers was significantly lower after 3 months. Based on the positive results on these outcome measures, together with a shorter length of stay in hospital and geriatric rehabilitation facility [20], we are recommend implementing the integrated care pathway in regular care. When implementing the pathway in regular care, it is important to keep monitoring the effects on patients and informal caregivers, but also on process related factors such as length of stay in hospital and in geriatric rehabilitation facility. It is also recommended to optimize the pathway elements which were not fully implemented according to plan, and to explore if all primary care providers in the Maastricht region are aware of the content of the integrated care pathway. Based on this exploration, targeted implementation strategies should be used for those primary care professionals who are unaware of its content or have not implemented it in daily practice. It is expected that this may prolong effects on patients and informal caregivers. Finally, more studies in the field of integrated care pathways in geriatric rehabilitation are needed to assess its impact on functional gains, discharge destination and length of stay in hospital and geriatric rehabilitation facility.

## Appendix

**Table 6:** Integrated care pathway for geriatric rehabilitation

Setting	No.	Care pathway element
Hospital	1	If the main treatment provider believes that the patient is eligible for geriatric rehabilitation, the discharge nurses of the hospital will be consulted. Preferably, this consultation takes place well in advance of discharge.
	2	Dismissal from the hospital is preceded by a triage by a discharge nurse. Information about the patient’s functional prognosis, endurability, teachability and trainability and the patient’s and informal caregiver’s needs and abilities needs to be gathered to make this triage decision.
	3	The triage is always performed under the responsibility of an elderly care physician from the geriatric rehabilitation facility. If the discharge nurse has doubts about the patient’s eligibility for geriatric rehabilitation, the elderly care physician should be consulted.
	4	Information about functional prognosis, endurability, teachability and trainability and needs and abilities of the patient should be gathered by consulting professionals in the hospital who have been involved in the patient’s care.
	5	The patient should always be asked about their needs and abilities and this should explicitly be taken into account when making the triage decision.
	6	The informal caregiver should (if applicable) be asked about their ability to provide informal care and this should explicitly be taken into account when making the triage decision.
	7	The discharge nurse should always provide oral and written information about geriatric rehabilitation to the patient and the informal caregiver.
	8	On the day the patient is discharged from the hospital, an up-to-date list of medications, a medical and nursing discharge summary and, if necessary, a discharge summary from allied health professionals should be available for the professionals in the geriatric rehabilitation facility.
Geriatric rehabilitation facility	9	In the cases where the patient discharge summaries are not available on the day the patient is admitted to the geriatric rehabilitation facility, professionals from the geriatric rehabilitation facility should contact the hospital directly.
	10	All patients with complex care needs admitted to the geriatric rehabilitation facility receive a systematic and multidisciplinary examination to determine which rehabilitation program is suitable for the patient.
	11	The patient’s rehabilitation program will be established in close consultation with patient and (if applicable) informal caregiver. The patient’s wishes and abilities and their informal caregiving situation will be taken into account when determining this program.
	12	Multidisciplinary meetings are organized at least twice during the patient’s stay.
	13	Patients and (if applicable) informal caregivers should always receive feedback on the issues discussed during the multidisciplinary meetings. In those cases where a modification to the patient’s rehabilitation program is desirable, this will be discussed with the patient and informal caregiver.
	14	Within two weeks after admission to the geriatric rehabilitation facility, the patient and (if applicable) informal caregiver will be informed about the patient’s provisional discharge date.
	15	The treatment intensity should be adjusted (decreased or increased) if this is required by the progress the patient is making.
	16	The provisional discharge date should be adjusted (decreased or increased) if this is required by the progress the patient is making.
	17	Well before discharge, the patient’s home situation should be mapped out by a physiotherapist or occupational therapist.
	18	After the home visit, advice should be given to the patient about required adjustments and aids in the home.
	19	The nurses in the geriatric rehabilitation facility should arrange home care prior to discharge of the patient.
	20	If the situation of the patient is complex, a professional of the home care organization will visit the geriatric rehabilitation facility for an intake.
	21	A professional of the home care organization will visit the geriatric rehabilitation facility for an intake if this is preferred by the patient.
	22	An up-to-date nursing discharge summary will be sent to the home care organization on the day of discharge.
	23	An up-to-date prescription for medication will be sent to the patient’s pharmacy on the day of discharge.
	24	An up-to-date discharge summary by allied health professionals will be given to the patient on the day of discharge.
	25	An up-to-date medical discharge summary and medication list will be sent to the patient’s general practitioner on the day of discharge.
	26	The discharge summary to the general practitioner includes information on the follow-up care advised.
Primary care	27	In those cases where the patient discharge summaries are not available to the primary care providers on the day the patient is discharged from the geriatric rehabilitation facility, professionals from the primary care organizations should directly contact the geriatric rehabilitation facility.
	28	Once the patient is discharged from the geriatric rehabilitation facility, the nurse practitioner or district nurse in primary care should act as the patient’s case manager.
All settings	29	A care pathway coordinator is appointed. The role of the care pathway coordinator is to act as a port of call for professionals involved in the pathway, to improve communication between professionals from different settings, improve continuity and coordinate care and to further streamline the pathway.
	30	At least twice per year, a meeting is organized between professionals from the hospital and from the geriatric rehabilitation facility who are involved in the triage process. The aim of this meeting is to evaluate whether or not the triage process, the medical discharge summaries and the transfer of patients between the hospital and the geriatric rehabilitation facility are satisfactory.
	31	At least once a year a meeting is organized between professionals from the geriatric rehabilitation facility and from primary care to evaluate the timing and quality of the medical discharge summaries and patient transfers.

## Abbreviations

ADL: Activities of daily living
CSAL: Cantril’s Self Anchoring Ladder
Erasmus iBMG: Erasmus Instituut Beleid & Management Gezondheidszorg
FAI: Frenchay Activities Index
IPA: Impact on Participation and Autonomy
KATZ-15: KATZ Index of independence in activities of daily living
RAND-36: RAND-36 item Health Survey
SRB-VAS: Self rated burden – visual analogue scale
